# Baseline Interleukin-6 in Sepsis: Mortality Risk Stratification and Survival Analysis in a Prospective Cohort

**DOI:** 10.3390/biomedicines14050990

**Published:** 2026-04-26

**Authors:** Raluca Terteşş, Lucian Cristian Petcu, Constantin Ionescu, Ionuţ Bulbuc, Anca Daniela Pînzaru, Bogdan Florentin Niţu, Lavinia-Carmen Daba, Elena Mocanu, Stela Halichidis, Nicolae Cârciumaru, Simona Claudia Cambrea

**Affiliations:** 1 Clinical Hospital of Infectious Diseases Constanta, Ferdinand Blvd. No. 100, 900709 Constanţa, Romaniacambrea.claudia@gmail.com (S.C.C.); 2 Faculty of Medicine, Ovidius University Constanta, Aleea Universității No. 1, Building B, 900470 Constanţa, Romaniai.bulbuc@gmail.com (I.B.); elena.mocanu@univ-ovidius.ro (E.M.);

**Keywords:** sepsis, biomarker, interleukin-6, prognosis, mortality

## Abstract

**Background/Objectives:** Sepsis is defined as life-threatening organ dysfunction caused by a dysregulated host response to infection. Identifying reliable biomarkers that reflect the underlying immune pathophysiology of sepsis and support early risk stratification remains a major clinical priority. This prospective study aimed to evaluate the prognostic value of interleukin-6 (IL-6) measured at ICU admission in patients with sepsis and septic shock. **Methods:** This prospective observational study included adult patients with sepsis and septic shock admitted to the Intensive Care Unit (ICU) of the Clinical Hospital of Infectious Diseases Constanța between 2021 and 2025. Receiver operating characteristic (ROC) curve analysis with DeLong comparisons, Kaplan–Meier survival analysis, and Cox proportional hazards regression modeling were performed to assess the association between baseline IL-6 levels, in-hospital mortality, and time to death. **Results:** Among the analyzed biomarkers, IL-6 demonstrated the highest discriminatory performance for in-hospital mortality (AUC = 0.956; 95% CI: 0.893–0.987; *p* < 0.0001). The optimal cut-off value (>135.14 pg/mL) yielded a sensitivity of 87.65% and specificity of 92.86% (Youden index = 0.805). However, despite this excellent discrimination between survivors and non-survivors, baseline IL-6 levels were not significantly associated with time-to-death in Cox proportional hazards regression analysis. **Conclusions:** Admission IL-6 showed excellent discriminatory performance for mortality risk stratification but was not associated with survival duration in time-to-event analyses. These findings suggest that IL-6 should be interpreted primarily as an early risk stratification biomarker rather than a predictor of survival duration in patients with sepsis.

## 1. Introduction

Sepsis is defined as life-threatening organ dysfunction caused by a dysregulated host response to infection [1]. In its most severe form, it may progress to septic shock, characterized by profound circulatory and metabolic abnormalities, leading to multiple organ failure and death [1]. The World Health Organization recognizes sepsis as a global health priority, with approximately 11 million sepsis-related deaths reported annually worldwide [2], prompting urgent calls for improved prevention, early diagnosis, and risk-adapted therapeutic strategies [3,4].

Despite advances in antimicrobial therapy and organ support, early identification of patients at high risk of adverse outcomes remains a major clinical challenge. Prognostic assessment currently relies largely on clinical severity scores such as SOFA and APACHE II, along with routine laboratory parameters, which may not fully capture the complexity and heterogeneity of the host inflammatory response [5]. Therefore, identifying reliable biomarkers that reflect the underlying immune pathophysiology of sepsis and allow early risk stratification remains of considerable clinical interest [6].

An ideal biomarker should be objectively measurable, reproducible, readily available, and capable of providing both diagnostic and prognostic information. Importantly, it should offer incremental value beyond established clinical scores and allow dynamic monitoring of treatment response [7,8]. Among candidate biomarkers, cytokines play a significant role, as they directly mediate and regulate the systemic inflammatory cascade characteristic of sepsis [9]. The release of pro-inflammatory cytokines, including IL-6, following the innate immune recognition of pathogen-associated molecular patterns (PAMPs) is a key mechanism in the host response to infection [10]. Therefore, circulating IL-6 levels represent a promising biomarker reflecting the dysregulated inflammatory response that characterizes the pathophysiology of sepsis. Previous studies have shown that serum concentrations of IL-6 increase significantly during sepsis and are associated with a higher risk of adverse outcomes, including septic shock and death [11,12].

Interleukin-6 (IL-6) is a pleiotropic cytokine with both pro- and anti-inflammatory properties and is rapidly released following infectious insult [13]. In healthy individuals, circulating IL-6 levels are typically low (<10 pg/mL), while in septic patients they increase rapidly within the first hours of infection onset and may reach markedly elevated concentrations [14]. Early studies showed significantly higher IL-6 levels in non-survivors compared to survivors [15,16], and subsequent investigations reported consistent associations between IL-6 concentrations, severity of organ dysfunction, and mortality risk.

Several observational studies have evaluated the prognostic significance of IL-6 in sepsis using survival modeling approaches. In a prospective cohort study, Song et al. [17] reported that elevated IL-6 levels at admission were associated with increased 28-day mortality. Similarly, Liu et al. [18] found that IL-6 retained independent prognostic significance in multivariable Cox proportional hazards models after adjustment for clinical and demographic variables. In patients with severe sepsis, Andaluz-Ojeda et al. [19] further showed that cytokine-based models incorporating IL-6 improved mortality prediction compared with clinical parameters alone. Although the reported discriminatory performance varies across cohorts, these findings collectively support the potential utility of IL-6 as an early marker for mortality risk stratification in patients with sepsis.

Few studies have directly compared IL-6 with established clinical severity scores and routinely available inflammatory markers within the same ICU population. Moreover, while many investigations have focused primarily on mortality classification, the relationship between baseline IL-6 levels and survival duration has not been consistently explored using both ROC-based discrimination and time-to-event modeling approaches within a unified analytical framework.

In this context, the present prospective study aimed to evaluate the prognostic role of IL-6 measured at ICU admission by: (i) assessing its discriminatory performance for in-hospital mortality using ROC analysis; (ii) directly comparing its predictive capacity with established clinical severity scores and routinely available inflammatory markers within the same cohort; and (iii) examining its association with survival duration using Cox proportional hazards modeling.

The novelty of this study lies not in reaffirming the association between IL-6 and mortality per se, but in providing a prospective head-to-head comparison between IL-6, established clinical severity scores, and routinely available biomarkers, thereby offering a more clinically contextualized evaluation of its potential contribution to early risk stratification in sepsis.

## 2. Materials and Methods

### 2.1. Study Design and Population

This prospective observational study was conducted at the Clinical Hospital of Infectious Diseases Constanța between 2021 and 2025. A total of 95 consecutive eligible adult patients diagnosed with sepsis or septic shock and admitted to the Intensive Care Unit (ICU) were included. Patients were eligible if they were ≥18 years old and met the Sepsis-3 diagnostic criteria (2016), defined as suspected or confirmed infection associated with an acute increase in Sequential Organ Failure Assessment (SOFA) score ≥ 2 points [1].

Exclusion criteria included:

(i) Incomplete medical records;
(ii) Preexisting hematological malignancies, solid organ malignancies (including metastatic disease), autoimmune disorders, HIV infection, or other known immunosuppressive conditions;
(iii) Death occurring within the first 24 h of ICU admission.

Although death within the first 24 h of ICU admission was predefined as an exclusion criterion, no patients met this criterion during the study period; therefore, it did not influence the final composition of the study cohort.

### 2.2. Data Collection

Demographic data (age and sex), clinical severity scores (SOFA and APACHE II), and laboratory results were extracted from electronic medical records.

Biomarkers analyzed at ICU admission (day 1) included:

Inflammatory markers: C-reactive protein (CRP), interleukin-6 (IL-6), erythrocyte sedimentation rate (ESR), serum ferritin, fibrinogen, and procalcitonin.

Hematological markers: White blood cell count, neutrophils, lymphocytes, and neutrophil-to-lymphocyte ratio (NLR).

Clinical severity scores: SOFA and APACHE II.

All biological parameters were obtained from routine blood tests performed at admission according to institutional protocols.

Serum IL-6 concentrations were measured using an enzyme-linked immunosorbent assay (ELISA) kit (IL E-3200; LDN Labor Diagnostika Nord GmbH & Co. KG, Nordhorn, Germany) according to the manufacturer’s instructions. Absorbance was measured at 450 nm using a Gemini XPS microplate reader (Molecular Devices, Sunnyvale, CA, USA). Results were expressed in pg/mL, with a detection limit of 2 pg/mL.

C-reactive protein (CRP) concentrations were determined using an immunoturbidimetric method on an automated chemistry analyzer (Mindray BS-480; Mindray Bio-Medical Electronics Co., Shenzhen, China), with a reference range of 0–8 mg/L.

Serum ferritin concentrations were measured using an enzyme-linked fluorescent assay (ELFA) on an automated immunoassay analyzer (mini VIDAS; bioMérieux, Marcy-l’Étoile, France), with a reference range of 10–120 ng/mL.

Serum procalcitonin (PCT) concentrations were measured using a chemiluminescence immunoassay (CLIA) on an automated immunoassay analyzer (Mindray CL-1200; Mindray Bio-Medical Electronics Co., Shenzhen, China), with a lower detection limit of 0.05 ng/mL.

Erythrocyte sedimentation rate (ESR) was assessed using the standardized Westergren method with a HumaRate device (Human GmbH, Wiesbaden, Germany), with a reference range of 3–13 mm/h.

Plasma fibrinogen concentrations were measured using an optical method on an automated coagulation analyzer (Sysmex CA-600; Sysmex Corporation, Kobe, Japan). The reference range was 140–360 mg/dL.

Complete blood count (CBC), including leukocyte, neutrophil, and lymphocyte counts, as well as the automatically calculated neutrophil-to-lymphocyte ratio (NLR), was performed at admission using an automated hematology analyzer (Mindray BC-6200; Mindray Bio-Medical Electronics Co., Shenzhen, China).

All laboratory analyses were conducted in the institutional clinical laboratory and subjected to internal and external quality control procedures.

### 2.3. Outcome

The primary outcome of the study was in-hospital mortality. Patients were stratified into two groups according to survival status at hospital discharge: survivors (discharged alive) and non-survivors (patients who died during hospitalization). For time-to-event analyses, survival time was defined as the number of days from ICU admission to death. Patients discharged alive were treated as censored at the date of hospital discharge.

### 2.4. Statistical Analysis

Continuous variables were expressed as median (interquartile range, IQR) and compared using the Mann–Whitney U test. Categorical variables were expressed as counts and percentages and compared using the chi-square test.

The discriminatory performance of baseline biomarkers for in-hospital mortality was assessed using receiver operating characteristic (ROC) curve analysis. The area under the curve (AUC) was calculated with 95% confidence intervals, and the optimal cut-off value was determined using the Youden index. Comparisons between AUC values were performed using the DeLong test.

Time-to-event analysis was conducted to evaluate the association between baseline IL-6 levels and time to death. Kaplan–Meier survival curves were constructed and compared using the log-rank test.

Cox proportional hazards regression analysis was performed to estimate hazard ratios (HRs) with 95% confidence intervals. IL-6 was analyzed both as a continuous variable and after logarithmic transformation to account for its right-skewed distribution.

A two-sided *p*-value < 0.05 was considered statistically significant.

All statistical analyses were performed using MedCalc version 14.8.1 (MedCalc Software Ltd., Ostend, Belgium).

### 2.5. Ethics

The study protocol was approved by the Ethics Committee of the Clinical Hospital of Infectious Diseases Constanța, in accordance with the Declaration of Helsinki approval code: No. 12, approval date: 1 September 2021, and informed consent was obtained from all participants or their legal representatives.

## 3. Results

### 3.1. Baseline Characteristics of the Study Population Stratified According to Clinical Outcome

Among the 95 patients included in the study, 81 (85.26%) died during hospitalization and 14 (14.74%) survived. Non-survivors tended to be older than survivors (66.96 ± 11.38 vs. 60.93 ± 14.18 years), although this difference did not reach statistical significance (*p* = 0.081) (Table 1).

Sex distribution was comparable between groups, with male patients representing 62.96% of non-survivors and 50.0% of survivors, without a statistically significant association with mortality (*p* = 0.358) (Table 1). Overall, baseline demographic characteristics were similar between survivors and non-survivors.

Disease severity at ICU admission was assessed using the SOFA and APACHE II scores. Non-survivors had significantly higher SOFA scores than survivors (median 5.0 [IQR 4.0–6.0] vs. 3.5 [IQR 2.75–4.0], *p* < 0.001) (Table 1), indicating greater organ dysfunction at admission. In contrast, APACHE II scores did not differ significantly between groups (median 10.0 [IQR 8.0–13.0] vs. 9.5 [IQR 8.75–12.0], *p* = 0.642).

Among the analyzed biomarkers, admission IL-6 levels were significantly higher in non-survivors than in survivors (median 198.76 vs. 113.35 pg/mL, *p* < 0.001). Procalcitonin and ferritin levels also differed significantly between groups (*p* = 0.007 and *p* = 0.024, respectively).

No significant differences were observed between survivors and non-survivors for CRP, ESR, fibrinogen, leukocyte count, neutrophil count, lymphocyte count, or NLR (all *p* > 0.05).

### 3.2. ROC Analysis

**Figure 1:**
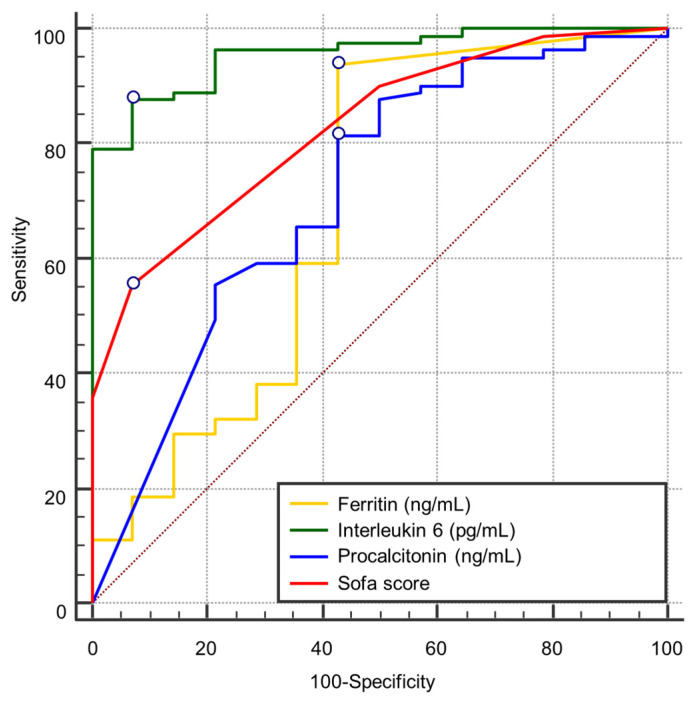
Receiver operating characteristic (ROC) curve analysis comparing the discriminatory performance of IL-6, ferritin, procalcitonin, and SOFA score for predicting in-hospital mortality. The dotted diagonal line represents the line of no discrimination (AUC = 0.5).

Receiver operating characteristic (ROC) curve analysis identified four admission variables with statistically significant predictive value for in-hospital mortality in septic patients: interleukin-6 (IL-6), SOFA score, procalcitonin, and ferritin (Figure 1). The discriminatory performance of each variable was evaluated using the area under the curve (AUC), sensitivity, specificity, Youden index, and optimal cut-off values.

Among all biomarkers, IL-6 demonstrated the highest prognostic performance, with an AUC of 0.956 (95% CI: 0.893–0.987, *p* < 0.0001). The optimal cut-off value (>135.14 pg/mL) yielded a sensitivity of 87.65% and specificity of 92.86%, corresponding to a Youden index of 0.805 (Table 2).

The SOFA score at admission showed good discriminatory performance, with an AUC of 0.828 (95% CI: 0.736–0.897, *p* < 0.0001). A cut-off value > 4 provided high specificity (92.86%) but lower sensitivity (55.56%) (Table 3).

Serum procalcitonin demonstrated moderate discriminatory ability, with an AUC of 0.714 (95% CI: 0.612–0.802, *p* = 0.0083). The optimal threshold (≤0.79 ng/mL) yielded a sensitivity of 81.48% and specificity of 57.14% (Table 2).

Serum ferritin showed borderline statistical significance, with an AUC of 0.689 (95% CI: 0.586–0.780, *p* = 0.0482). The optimal cut-off value (≤1147.00 ng/mL) provided high sensitivity (93.83%) but limited specificity (57.14%) (Table 2).

The remaining variables (leukocyte count, neutrophils, lymphocytes, CRP, ESR, fibrinogen, NLR, and APACHE II score) did not reach statistical significance (all *p* > 0.05), indicating limited discriminatory performance for mortality prediction when analyzed individually at admission (Table 3 and Table 4).

Comparative analysis using the DeLong test demonstrated that the AUC of IL-6 was significantly higher than those of procalcitonin (difference = 0.242, *p* = 0.0027), ferritin (difference = 0.267, *p* = 0.0047), and SOFA score (difference = 0.128, *p* = 0.0056), further supporting the superior predictive performance of IL-6 (Table 5).

### 3.3. Kaplan–Meier Survival Analysis

Kaplan–Meier survival analysis was performed to estimate in-hospital survival time in the study cohort. Of the 95 included patients, 81 (85.26%) reached the study endpoint (death), while 14 (14.74%) were discharged alive.

The mean survival time was 22.43 days (standard error = 1.879), with a 95% confidence interval (CI) of 18.75–26.11 days. The median survival time, defined as the time at which 50% of patients experienced the event, was 17.00 days (95% CI: 15.00–20.00 days) (Table 6).

**Figure 2:**
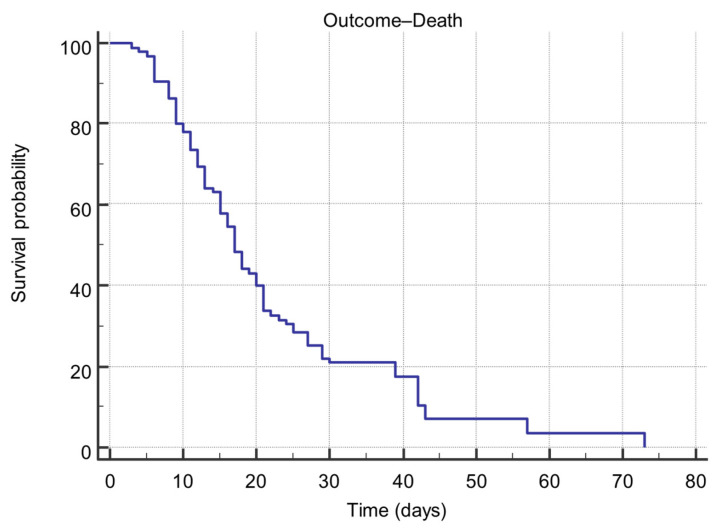
Kaplan–Meier estimate of overall survival in the study cohort. Survival time was defined as the number of days from ICU admission to death. The median survival time was 17 days (95% CI: 15–20 days), and the mean survival time was 22.43 days (95% CI: 18.75–26.11 days).

The Kaplan–Meier survival curve showed a progressive decline in survival probability over time. The estimated survival probability was 77.9% on day 10, decreasing to 48.4% on day 17 and reaching 0% by day 73 (Figure 2). A marked decline in survival probability between days 10 and 25 suggests a critical deterioration phase during the second and third weeks of hospitalization.

### 3.4. Survival Analysis Based on Baseline IL-6

Survival analyses were performed to evaluate the association between baseline IL-6 levels and time to death in the study cohort. Kaplan–Meier survival curves and Cox proportional hazards regression models were generated using MedCalc version 14.8.1.

#### 3.4.1. Cox Proportional-Hazards Regression

Cox regression analysis, including IL-6 as a continuous variable, showed no significant association between admission IL-6 levels and mortality hazard. The model did not significantly improve fit compared with the null model (χ^2^ = 0.0365, *p* = 0.8485) (Table 7). The regression coefficient for IL-6 was not significant (b = −0.0002521, *p* = 0.8485), with a hazard ratio (HR) of 0.9997 (95% CI: 0.9972–1.0023).

These findings indicate that baseline IL-6 values were not associated with time to death in this cohort.

#### 3.4.2. Cox Regression Using Log-Transformed IL-6

Because IL-6 values showed a skewed distribution, a secondary Cox regression model using log-transformed IL-6 [ln (IL-6)] was performed. Similar results were obtained, with no significant improvement in model fit (χ^2^ = 0.00889, *p* = 0.9249). The regression coefficient remained non-significant (b = −0.01021, *p* = 0.9250), and the hazard ratio remained close to unity (HR = 0.9898; 95% CI: 0.8013–1.2228).

Thus, logarithmic transformation did not change the absence of association between IL-6 and survival time.

#### 3.4.3. Survival Analysis Based on ROC-Derived IL-6 Cut-Off

**Figure 3:**
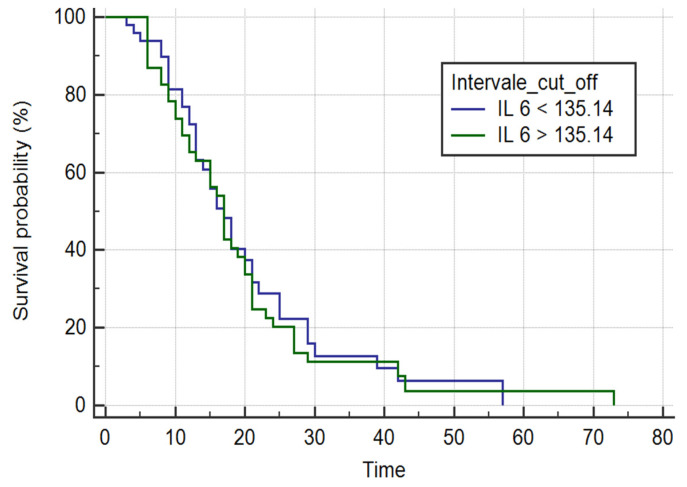
Kaplan–Meier survival curves stratified according to baseline IL-6 levels using the ROC-derived cut-off value (135.14 pg/mL). Survival time was defined as the number of days from ICU admission to death. Differences between groups were assessed using the log-rank test (*p* = 0.7306).

To evaluate potential risk stratification, patients were categorized according to the ROC-derived IL-6 cut-off value (135.14 pg/mL) (Figure 3). Kaplan–Meier analysis showed no significant difference between survival curves (log-rank χ^2^ = 0.1186, *p* = 0.7306). Median survival time was identical in both groups (17 days), and estimated hazard ratios remained close to 1 with wide confidence intervals.

Similarly, Cox regression using this dichotomized variable was not significant (χ^2^ = 0.110, *p* = 0.7402; HR = 1.0772, 95% CI: 0.6955–1.6681).

These findings indicate that stratification according to the ROC-based threshold did not discriminate time-to-event outcomes.

#### 3.4.4. Quartile-Based IL-6 Stratification

**Figure 4:**
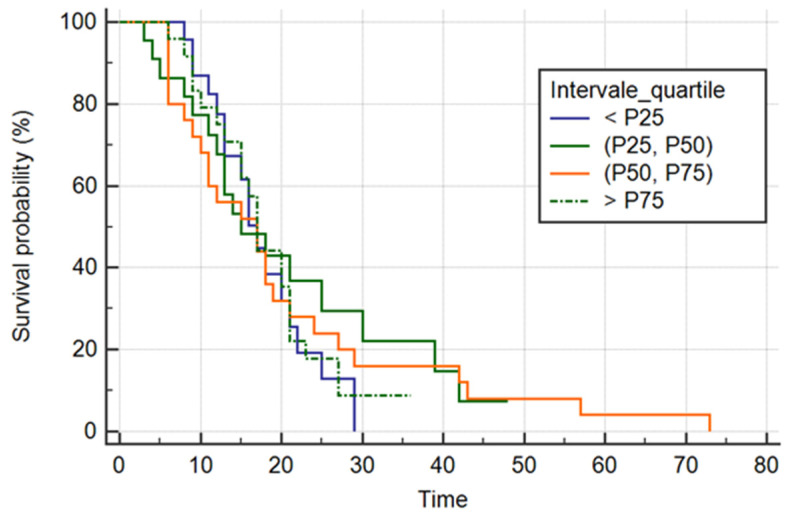
Kaplan–Meier survival curves stratified according to quartiles of baseline IL-6 levels. Survival time was defined as the number of days from ICU admission to death. Differences between groups were assessed using the log-rank test (*p* = 0.9457).

To further explore a possible dose–response relationship, IL-6 values were categorized into quartiles (P25, P50, P75) (Figure 4).

Kaplan–Meier analysis showed no significant differences between survival curves across quartiles (log-rank χ^2^ = 0.3735, *p* = 0.9457). Median survival times were similar across groups (approximately 17 days), and hazard ratios remained close to 1.

Consistently, Cox regression using quartile categories did not show a significant overall model fit (χ^2^ = 0.348, *p* = 0.9508), and none of the higher quartiles showed an increased hazard compared with the reference category (<P25).

Across all survival models evaluated—continuous IL-6, log-transformed IL-6, ROC-derived cut-off stratification, and quartile-based categorization—no significant association was observed between admission IL-6 levels and time to death.

Overall, survival analyses consistently showed that admission IL-6 values were not associated with time-to-event outcomes. Although IL-6 demonstrated excellent discriminatory performance between survivors and non-survivors in ROC analysis, baseline IL-6 levels did not predict the timing of death, supporting its interpretation as a risk-stratification biomarker rather than a predictor of survival duration.

## 4. Discussion

Early identification of high-risk patients with sepsis remains a major challenge in the management of critically ill patients [20,21]. Interleukin-6 (IL-6) has been extensively investigated as a prognostic biomarker in sepsis, with previous studies reporting moderate discriminatory performance for mortality prediction [22]. The existing literature consistently supports the prognostic relevance of IL-6 in septic populations [22].

In the present study, baseline IL-6 demonstrated excellent discriminatory ability between survivors and non-survivors in ROC analysis, with an AUC of 0.956 (95% CI: 0.893–0.987), high sensitivity (87.65%), and high specificity (92.86%) at a cut-off value of 135.14 pg/mL. This finding is consistent with previous reports identifying IL-6 as a key cytokine in both the pathogenesis and prognosis of sepsis. IL-6 is a central mediator of the dysregulated inflammatory response and contributes to the cytokine storm characteristic of severe sepsis and septic shock. Moreover, IL-6 signaling has been associated with endothelial activation, microvascular dysfunction, and subsequent organ failure in critically ill patients [23,24,25].

In a recent prospective study of patients with septic shock, Gamarra-Morales et al. [26] reported moderate discriminatory performance of baseline IL-6 for mortality prediction (AUC = 0.826, 95% CI: 0.659–0.994, *p* < 0.008). Similarly, Pettilä et al. [27] reported moderate discriminatory performance for IL-6 measured on day 2 (AUC = 0.799) in predicting hospital mortality in critically ill patients with suspected sepsis, whereas admission IL-6 did not show comparable predictive accuracy in their cohort. In contrast, baseline IL-6 in our cohort showed higher discriminatory performance (AUC = 0.956). This discrepancy may reflect differences in study design, mortality rates, disease severity distribution, and the relatively small proportion of survivors in our cohort, which may have amplified discrimination estimates. These findings highlight the context-dependent nature of IL-6 prognostic performance across septic populations.

The SOFA score at admission also demonstrated strong predictive performance for mortality (AUC = 0.828). Although its performance was inferior to that of IL-6 (*p* = 0.0056), the SOFA score remains a validated and widely used clinical tool for risk stratification in critically ill patients [28]. It is also endorsed in the Sepsis-3 definition for assessing organ dysfunction and predicting outcomes [29]. In our cohort, specificity was high (92.86%) at a cut-off value > 4, whereas sensitivity was moderate (55.56%), suggesting that the SOFA score alone may fail to identify a substantial proportion of high-risk patients when used in isolation. Previous studies have demonstrated that integrating the SOFA score with IL-6 improves prognostic accuracy for mortality prediction in patients with sepsis [30].

Procalcitonin, a biomarker widely used in sepsis management, showed moderate discriminatory performance (AUC = 0.714). Its sensitivity (81.48%) and specificity (57.14%) support its role as a complementary marker, particularly for assessing bacterial infection severity and guiding antibiotic stewardship. However, its performance remained significantly inferior to that of IL-6 (*p* = 0.0027), indicating that it should not be used as an isolated prognostic indicator [31].

In the present cohort, median procalcitonin levels were unexpectedly higher in survivors than in non-survivors. This finding may be explained by the heterogeneous etiology of sepsis in the study population. Procalcitonin levels typically increase in bacterial infections and remain lower in viral sepsis [32]. Therefore, differences in the distribution of infection types between outcome groups may have influenced baseline procalcitonin concentrations in this mixed cohort rather than reflecting mortality risk alone. These findings support the interpretation of procalcitonin as a complementary biomarker reflecting infection characteristics rather than an independent predictor of outcome.

Ferritin, an acute-phase reactant associated with hyperinflammation and macrophage activation, showed lower but statistically significant discriminatory performance (AUC = 0.689, *p* = 0.0482). Although its predictive ability was inferior to that of IL-6 and the SOFA score, elevated ferritin levels remain associated with poor outcomes in sepsis and septic shock, particularly in hyperferritinemic syndromes [33].

Other tested variables, including lymphocyte count, NLR, CRP, ESR, and fibrinogen, did not demonstrate significant discriminatory performance (AUC < 0.7, *p* > 0.05). These findings support a shift from traditional inflammatory markers toward cytokine-based or integrated multimarker approaches for prognostic assessment in sepsis.

Although admission IL-6 demonstrated excellent discrimination for in-hospital mortality in ROC analysis, it was not significantly associated with time to death in Cox proportional hazards models. Across all survival models evaluated, no significant association was identified between baseline IL-6 levels and time-to-event outcomes.

This apparent discrepancy likely reflects the conceptual difference between binary outcome discrimination and time-to-event modeling. ROC analysis evaluates the ability of a biomarker to distinguish survivors from non-survivors, whereas Cox regression assesses whether the biomarker influences survival duration. In our cohort, IL-6 appeared to function primarily as a risk-stratification biomarker rather than a predictor of survival duration. Similar variability in survival modeling results has been reported in other sepsis cohorts, where IL-6 was associated with mortality status but showed inconsistent associations with survival duration depending on analytical strategy and adjustment variables [17,18].

The high in-hospital mortality rate and the relatively small proportion of survivors may have contributed to amplified discrimination estimates in ROC analysis, whereas time-to-event models primarily captured variability in survival duration. In our cohort, survival times were clustered within a relatively narrow interval, which may explain why baseline IL-6 differentiated mortality status but did not predict the timing of death.

From a biological perspective, IL-6 reflects the magnitude of the early inflammatory response at admission. Elevated levels may identify patients at higher overall risk of adverse outcomes; however, once severe organ dysfunction is established, the timing of death is likely influenced by multiple downstream pathophysiological mechanisms beyond the initial cytokine response. The pathophysiological role of IL-6 in the dysregulated host response to infection supports its association with disease severity and organ dysfunction [23,24], although its temporal dynamics during critical illness may vary substantially between patients.

Previous studies have reported significant associations between elevated IL-6 levels and mortality in multivariable regression models. In a prospective cohort of septic patients, IL-6 retained independent prognostic significance after adjustment for clinical and demographic variables [18]. Similarly, cytokine-based models incorporating IL-6 improved mortality prediction in severe sepsis [19]. In another prospective study, Andaluz-Ojeda et al. demonstrated that IL-6 remained independently associated with increased mortality after adjustment for APACHE II score. Furthermore, a combined cytokine score incorporating IL-6, IL-8, and IL-10 showed an even stronger association with mortality in multivariable Cox models [19]. These findings suggest that IL-6 contributes independently to mortality risk stratification, particularly when integrated into composite inflammatory scores.

Kaplan–Meier survival analysis has also been used to evaluate the prognostic value of IL-6 in septic populations [17]. In one cohort, patients stratified according to an optimal IL-6 cut-off (≥348.9 pg/mL) showed significantly reduced 28-day survival compared with those below this threshold (log-rank *p* = 0.008) [17]. However, reported effect sizes and levels of statistical significance vary across studies, likely reflecting differences in patient characteristics, timing of biomarker measurement, assay platforms, and statistical modeling strategies.

From a clinical perspective, admission IL-6 levels may support early risk stratification and facilitate the identification of high-risk patients with a pronounced inflammatory burden who may require closer monitoring and more intensive supportive management. However, consistent with previous studies and meta-analyses, IL-6 appears to function primarily as a marker of global disease severity rather than as a reliable predictor of survival duration, supporting its use within integrated multimarker risk-assessment strategies rather than as a standalone time-to-event prognostic indicator.

Previous cohort studies have demonstrated that admission IL-6 levels correlate strongly with established severity scores such as APACHE II and SOFA (r ≈ 0.68–0.72), further supporting its role as a marker of global disease severity and early inflammatory burden rather than a determinant of survival timing [34]. Evidence from multimarker prognostic models also indicates that the predictive performance of IL-6 improves substantially when combined with other biomarkers such as lactate and procalcitonin [35]. For example, in a cohort of 367 patients diagnosed with sepsis according to Sepsis-3 criteria, models incorporating serial IL-6 together with lactate and procalcitonin achieved strong discrimination for 28-day mortality, supporting the role of IL-6 within integrated risk-stratification strategies rather than as an isolated predictor of outcome timing [35].

Furthermore, a recent meta-analysis including 31 studies and 4566 patients demonstrated that baseline IL-6 shows moderate-to-good discriminatory performance for short-term mortality (pooled AUROC 0.701), despite inconsistent independent associations in regression-based models, reinforcing its value primarily as a complementary admission-phase prognostic biomarker [22].

## 5. Conclusions

In this prospective ICU cohort, admission IL-6 demonstrated excellent discriminatory performance for mortality risk stratification but was not associated with survival duration in time-to-event analyses. These findings suggest that IL-6 primarily functions as a risk-stratification biomarker rather than as a predictor of survival duration.

Elevated IL-6 levels reflect the intensity of the early inflammatory response and may identify patients at increased overall risk; however, the temporal course of fatal outcomes in sepsis is likely influenced by multiple downstream pathophysiological mechanisms beyond the initial cytokine surge.

Therefore, admission IL-6 should be interpreted as a complementary biomarker that may enhance early mortality risk stratification when integrated with established severity scores and other prognostic biomarkers reported in the literature, supporting its role within multimarker risk-assessment strategies rather than as a standalone time-to-event prognostic tool.

Further multicenter studies with larger sample sizes and serial cytokine measurements are warranted to clarify the incremental prognostic value of IL-6 within integrated survival modeling frameworks.

This study has several limitations that should be acknowledged.

First, the single-center design and relatively limited sample size may restrict the generalizability of the findings. Although the number of events was high, the proportion of survivors was small, which may have influenced discrimination estimates and limited the stability of certain statistical comparisons.

Second, the high in-hospital mortality rate and the clustering of survival times within a relatively narrow time window (median 17 days) may have reduced the sensitivity of time-to-event models to detect hazard gradients. Consequently, while IL-6 showed strong discriminatory performance for mortality classification, its lack of association with survival duration should be interpreted within the context of limited temporal dispersion of events.

Third, IL-6 was measured at a single time point at ICU admission. Dynamic changes in cytokine levels over time may provide additional prognostic information that was not captured in the present analysis.

Fourth, the ROC-derived cut-off value was data-driven and internally derived from the same cohort, without external validation. This may lead to overestimation of discriminatory performance, particularly given the relatively small number of survivors. Independent validation in larger multicenter cohorts is warranted.

Finally, although several inflammatory and clinical severity parameters were evaluated, residual confounding cannot be excluded, and the prognostic performance of IL-6 may differ across patient populations, assay platforms, and therapeutic settings.

## Figures and Tables

**Table 1:** Baseline demographic, clinical, and laboratory characteristics of survivors and non-survivors.

Variable	Survivors (14)	Non-Survivors (81)	*p*-Value
Age (years), mean ± SD	60.93 ± 14.18	66.96 ± 11.38	0.081
Sex (male), n (%)	7 (50.0%)	51 (62.96%)	0.358
SOFA score, median (IQR)	3.50 (2.75–4.00)	5.00 (4.00–6.00)	0.001
APACHE II score, median (IQR)	9.50 (8.75–12.00)	10.00 (8.00–13.00)	0.642
IL-6 (pg/mL), median (IQR)	113.35 (24.07–119.52)	198.76 (154.05–219.56)	<0.001
CRP (mg/L), median (IQR)	131.00 (48.25–237.75)	125.00 (64.00–196.00)	0.962
Procalcitonin (ng/mL), median (IQR)	1.47 (0.10–6.34)	0.07 (0.05–0.07)	0.007
Ferritin (ng/mL), median (IQR)	1200.00 (718.79–1200.00)	819.65 (687.53–996.91)	0.024
ESR (mm/h), median (IQR)	54.00 (33.25–77.50)	60.00 (37.00–80.00)	0.729
Fibrinogen (mg/dL), median (IQR)	523.00 (385.25–789.50)	585.00 (462.50–756.50)	0.603
Leukocytes (×10^3^ cells/µL), median (IQR)	7790.00 (4992.50–13,720.00)	7180.00 (5375.00–10,710.00)	0.721
Neutrophils (×10^3^ cells/µL), median (IQR)	5810.00 (3687.50–12,752.75)	6310.00 (3995.00–9700.00)	0.992
Lymphocytes (×10^3^ cells/µL), median (IQR)	585.00 (487.50–892.50)	660.00 (465.00–950.00)	0.505
NLR, median (IQR)	11.49 (4.55–24.42)	10.51 (5.70–15.55)	0.324

Data are presented as mean ± standard deviation (SD) or median (interquartile range, IQR). Continuous variables were compared using the Mann–Whitney U test, and categorical variables using the chi-square test. Abbreviations: SOFA = Sequential Organ Failure Assessment; APACHE II = Acute Physiology and Chronic Health Evaluation II; IL-6 = interleukin-6; CRP = C-reactive protein; ESR = erythrocyte sedimentation rate; NLR = neutrophil-to-lymphocyte ratio.

**Table 2:** ROC curve analysis of inflammatory biomarkers for predicting in-hospital mortality.

Biomarker	AUC	SE	95% CI for AUC	z	p	J	Xk	Se (%)	Sp (%)
Ferritin (ng/mL)	0.689	0.0962	0.586 to 0.780	1.975	0.0482	0.5097	≤1147.00	93.83	57.14
IL-6 (pg/mL)	0.956	0.0211	0.893 to 0.987	21.646	<0.0001	0.8051	>135.14	87.65	92.86
CRP (mg/L)	0.504	0.0990	0.399 to 0.608	0.040	0.9680	0.1649	>304.00	4.94	78.57
ESR (mm/h)	0.529	0.0907	0.424 to 0.632	0.321	0.7483	0.2037	>48.00	70.37	50.00
Fibrinogen (mg/dL)	0.544	0.0922	0.438 to 0.646	0.474	0.6358	0.1914	>500.00	69.14	50.00
Procalcitonin (ng/mL)	0.714	0.0812	0.612 to 0.802	2.640	0.0083	0.3862	≤0.79	81.48	57.14

**Table 3:** ROC curve analysis of clinical severity scores for predicting in-hospital mortality.

Clinical Severity Score	AUC	SE	95% CI for AUC	z	p	J	X_k_	Se (%)	Sp (%)
SOFA score	0.828	0.0489	0.736 to 0.897	6.702	<0.0001	0.4841	>4.00	55.56	92.86
APACHE II score	0.539	0.0798	0.433 to 0.642	0.486	0.6268	0.1464	>10.00	43.21	71.43

**Table 4:** ROC curve analysis of hematological biomarkers for predicting in-hospital mortality.

Biomarker	AUC	SE	95% CI for AUC	z	p	J	Xk	Se (%)	Sp (%)
Leucocytes (×10^3^ cells/µL)	0.530	0.0892	0.425 to 0.633	0.336	0.7368	0.1623	≤13,450.00	87.65	28.57
Neutrophiles (×10^3^ cells/µL)	0.501	0.0926	0.396 to 0.605	0.010	0.9924	0.1623	≤11,290.00	87.65	28.57
Lymphocytes (×10^3^ cells/µL)	0.556	0.0828	0.450 to 0.658	0.676	0.4988	0.1834	>700.00	46.91	71.43
NLR	0.583	0.0942	0.477 to 0.683	0.880	0.3789	0.2460	≤18.37	88.89	35.71

AUC = area under the ROC curve; SE = standard error; z = test statistic; p = associated probability; J = Youden index (maximum vertical distance between the ROC curve and the diagonal reference line); X_k_ = cut-off value; Se (%) = sensitivity; Sp (%) = specificity.

**Table 5:** Comparison of ROC curve AUC values using the DeLong test in predicting in-hospital mortality.

Delong Test	Standard Error	95% Confidence Interval	*p*-Value
IL-6 (pg/mL) vs. SOFA score	0.0463	0.0375 to 0.219	0.0056
IL-6 (pg/mL) vs. Procalcitonin (ng/mL)	0.0805	0.0839 to 0.399	0.0027
IL-6 (pg/mL) vs. Ferritin (ng/mL)	0.0946	0.0818 to 0.453	0.0047
Ferritin (ng/mL) vs. Procalcitonin (ng/mL)	0.115	−0.200 to 0.251	0.8241
SOFA score vs. Ferritin (ng/mL)	0.0954	−0.0482 to 0.326	0.1456
SOFA score vs. Procalcitonin (ng/mL)	0.0789	−0.0413 to 0.268	0.1509

**Table 6:** Kaplan–Meier estimates of overall survival time from ICU admission to death in the study cohort.

Mean	SE	95% CI for the Mean	Median	95% CI for the Median
22.428	1.879	18.745 to 26.111	17.000	15.000 to 20.000

Survival time was defined as the number of days from ICU admission to death. CI = confidence interval; SE = standard error.

**Table 7:** Cox proportional hazards regression analysis evaluating the association between baseline IL-6 levels and time to death.

Model	Hazard Ratio (HR)	95% CI	*p*-Value
IL-6 (continuous, pg/mL)	0.9997	0.9972–1.0023	0.848
Log-transformed IL-6	0.9898	0.8013–1.2228	0.925
IL-6 ≥ 135.14 pg/mL (ROC-derived cut-off)	1.077	0.695–1.668	0.740

HR = hazard ratio; CI = confidence interval. Survival time was defined as the number of days from ICU admission to death.

## Data Availability

The original contributions presented in this study are included in the article. Further inquiries can be directed to the corresponding author.

## Abbreviations

IL-6	Interleukin 6
PAMPs	Pathogen-associated molecular patterns
ICU	Intensive care unit
ALC	Absolute lymphocyte count
NLR	Neutrophil-to-lymphocyte ratio
SOFA	Sequential organ failure score
ESR	Erythrocyte sedimentation rate
CRP	C-reactive protein
APACHE II	Acute Physiology and Chronic Health Evaluation II
PCT	Procalcitonin
